# MicroRNAs in Pediatric Primary Hypertension: Promising Biomarkers or Context-Dependent Regulators?—A Narrative Review

**DOI:** 10.3390/biom16050636

**Published:** 2026-04-24

**Authors:** Michał Szyszka, Piotr Skrzypczyk

**Affiliations:** 1 Department of Pediatrics and Nephrology, Doctoral School, Medical University of Warsaw, 02-091 Warsaw, Poland; michal.szyszka@wum.edu.pl; 2 Department of Pediatrics and Nephrology, Medical University of Warsaw, 02-091 Warsaw, Poland

**Keywords:** microRNA, epigenetic factors, primary hypertension, hypertension-mediated organ damage, biomarker, children

## Abstract

Hypertension affects approximately 1.4 billion people worldwide. It is worth noting that it is also found in approximately 4% of children, with about half of pediatric patients having primary hypertension. Despite its widespread prevalence, the pathogenesis of primary hypertension remains poorly understood. Among the many hypotheses, research into epigenetic factors, primarily microRNA, has gained momentum in recent years. The role of microRNA is to bind to mRNA and thus influence protein translation. It is postulated that the main sites where microRNA influences blood pressure are the vascular wall, vascular smooth muscle cells, and the kidney. Many microRNAs have been identified whose importance in primary hypertension development has been demonstrated in experimental and human studies. This article discusses in detail the significance of microRNA-16, -21, -27a, -27b, -133a, and -145. It presents the current state of knowledge, as well as the challenges, limitations, and uncertainties regarding the use of these fascinating molecules as biomarkers and in potential therapy. MicroRNAs are an extremely promising area of research into the pathogenesis of primary hypertension. However, we still do not know whether they will prove to be a promising biomarker of primary hypertension.

## 1. Introduction

### 1.1. The Definition, Epidemiology and Etiology of Hypertension in the Developmental Period

The European Society of Hypertension (ESH) and the HyperChildNET group use age-specific criteria to define pediatric arterial hypertension. In children aged 0–15 years, blood pressure is assessed using percentile charts; in adolescents aged 16–17 years, however, adult thresholds are applied [1,2]. Arterial hypertension is diagnosed when systolic and/or diastolic blood pressure is at or above the 95th percentile based on at least three separate measurements, or at or above 140/90 mmHg in adolescents. Both the European and American (American Academy of Pediatrics) guidelines define pediatric hypertension using age-, sex- and height-specific percentiles, which distinguishes it fundamentally from adult hypertension [3]. While these approaches are largely consistent, the American guidelines apply lower thresholds (≥130/80 mmHg) from the age of 13 years onwards and are based on reference values derived from children of normal weight. In contrast, European reference data are based on earlier population cohorts that did not explicitly exclude overweight and obese individuals.

According to estimates by the World Health Organization, AH occurs in 1.4 billion people aged 30–79. An analysis of our local and national data from 2018 to 2022 indicates that the number of patients with AH in Poland is stable and amounts to about 11 million—this disease affects about 35% of adult Poles [4]. However, population-based studies, such as the WOBASZ II survey, suggest a higher prevalence of approximately 42.7%, reflecting the burden of both diagnosed and undiagnosed hypertension [5].

In the literature, the percentage of patients with hypertension in the developmental period varies greatly and ranges from one to several percent, depending on the study and measurement methodology. A 2019 meta-analysis of 47 publications and 186,630 participants, published in JAMA Pediatrics, reports a 4% prevalence of AH in the developmental age group. The most common in developmental age is isolated systolic hypertension (1.5% of the population, 37.5% of children with AH). In the cited meta-analysis, there were no differences in AH incidence between boys and girls, nor were there differences by age. Still, it was shown that AH was most common in children with obesity, in whom it occurred with a frequency of 15.27%, followed by overweight children (4.99%), and least often in children with normal body weight (1.90%) [6].

Data from other epidemiological studies indicate an increasing incidence of hypertension with the age of the children studied. The disease is infrequent in infancy and early childhood, but during puberty its prevalence rises substantially, mostly among adolescent boys. Polish data indicate that AH affects 9% of people aged 18, including as many as 16% of males, which is the same as the rate of AH in the third decade of life [7]. Traditionally, it was believed that secondary hypertension dominates in the developmental age, unlike adults. However, large epidemiological data indicate that primary hypertension (pHTN) may occur in up to half of pediatric patients with AH and can be diagnosed in children in the first decade of life, especially those burdened with risk factors such as obesity and a positive family history [8,9]. In a recently published analysis of 2008 patients of one tertiary Polish center, out of 1260 patients with confirmed hypertension, pHTN was found in 49.3% of children, and pHTN secondary in the remaining 50.7% [10]. There is not the slightest doubt that pHTN is by far the most common etiology of AH in adolescence. The likelihood of pHTN is greater among males, those aged ≥ 12.5 years, overweight and obese children, and children with hyperuricemia [10]. In 15–20% of adolescents with pHTN, metabolic syndrome is diagnosed [11]. Although pediatric pHTN shares several pathophysiological mechanisms with adult hypertension, including genetic predisposition and neurohormonal dysregulation, lifestyle-related factors such as obesity, poor dietary habits, and physical inactivity appear to play an even more prominent role in the pediatric population.

In summary, it should be stated that taking into account the above epidemiological data, hypertension, and especially pHTN, is a significant health issue in the population of children and adolescents.

### 1.2. The Underlying Factors Contributing to the Development of Primary Hypertension

The pathogenesis of pHTN in both adults and children is multifactorial and unclear. Mean arterial pressure (MAP) is the product of cardiac output, which in turn is determined by stroke volume and heart rate, and peripheral (arterial) resistance. Each of these elements is strictly regulated by physiological mechanisms that ensure the normal, laminar flow of blood ejected from the left ventricle during contraction to all tissues and organs [12]. The classic two theories of hypertension development are Guyton’s hypothesis, assuming that pHTN is the result of increased blood volume (and thus cardiac output) due to an increase in sodium renal reuptake (the so-called increased renal threshold for sodium), and the Folkow hypothesis, indicating an increase in arterial wall tension (peripheral resistance) as the cause of hypertension. Rather, current concepts assume the involvement of multiple factors that dynamically increase each of these elements, thereby raising blood pressure (the so-called modified Page mosaic). According to the current knowledge, the main mechanisms involved in the pathogenesis of primary hypertension in children and adolescents were described in Table 1.

### 1.3. Epigenetic Factors in the Pathophysiology of Primary Hypertension

Epigenetic mechanisms regulate human homeostasis by influencing the expression of various genes. These mechanisms respond swiftly to environmental and health-related changes, regulating gene expression or cellular processes independently of DNA sequence changes.

**Figure 1:**
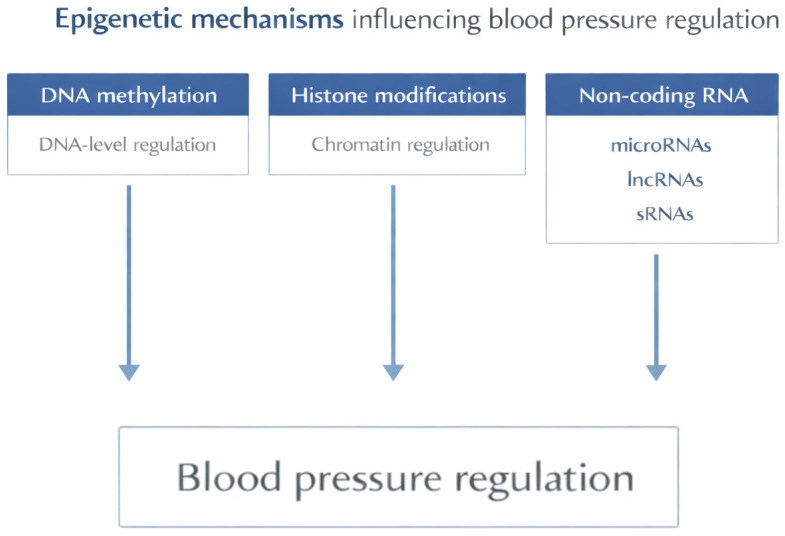
Epigenetic mechanisms influencing blood pressure regulation.

There are three main proposed epigenetic mechanisms influencing blood pressure regulation (Figure 1). They are described briefly below.

DNA methylation is the first epigenetic mechanism discovered. DNA chains are methylated mainly at cytosine (5-methylcytosine is formed) in cytosine-guanine dinucleotides (CpG). Human DNA is methylated non-symmetrically, with some areas heavily methylated. These DNA regions are transcriptionally inactive (heterochromatin). Conversely, some other DNA fragments are variably methylated depending on various exogenous and endogenous factors, such as diet and physical exercise. Generally, when promoter regions are heavily methylated, the gene is silent and not transcribed [32,33,34]. Recently, another methylation mark, N6-methyladenine (6mA), was discovered [33]. The significance of various factors, e.g., high-salt diet, and the level of methylation was unmasked in a mouse model [32]. Also, some human studies indicate a role for DNA methylation in the development of arterial hypertension [35,36]. Interestingly, in the aforementioned Indian study, methylation status differed between hypertensives with controlled and uncontrolled hypertension, and folate concentration seemed to influence methylation levels [35].

Histones are small, positively charged proteins that pack negatively charged DNA into nucleosomes, finally forming chromatin. Histone modifications influence gene activation or silencing. There are many forms of histone modifications—mainly amino acid modifications, e.g., lysine acetylation, lysine or arginine methylation, or serine or threonine phosphorylation, or larger alterations, i.e., histone protein sumoylation and ubiquitylation [37,38]. Human studies have shown a link between hypertension and the activity of histone-modifying enzymes, such as histone deacetylases (HDACs). Furthermore, in an animal model, treatment with HDAC inhibitors lowered blood pressure [37]. Lysine demethylation of histones at the TWIST1 promoter in macrophages was found to induce instability of the atherosclerotic plaque in another animal model [39].

The discovery of non-coding RNAs has opened an entirely new field of knowledge regarding the post-transcriptional regulation of gene expression. Depending on their length, non-coding RNAs are classified as short (<200 nt) and long (>200 nt). We currently know of more than 10 types of non-coding RNAs. These molecules are crucial for regulation, including basic housekeeping functions (ribosomal RNA—rRNA, transfer RNA—tRNA) and intricate gene regulation (long non-coding RNA–lncRNA and microRNA). They are implicated in numerous diseases, including cardiovascular diseases [40,41]. LncRNAs are transcripts > 200 nt, including molecules called Xist and HOTAIR, which regulate gene expression, chromatin remodeling, and epigenetic machinery. Animal models have shown that lncRNAs are involved in multiple processes involved in blood pressure regulation, including vascular smooth muscle cell (VSMC) proliferation and nitric oxide production [42]. It has recently been demonstrated that the lncRNA MALAT1 plays a role in the development of salt-induced hypertension, which opens up possibilities for targeting this RNA as a potential approach to the prevention and treatment of hypertension [43].

The three principal epigenetic mechanisms—DNA methylation, histone modifications, and non-coding RNAs—contribute to the regulation of gene expression without altering the DNA sequence. These processes collectively influence pathways involved in blood pressure control. DNA—deoxyribonucleic acid; RNA—ribonucleic acid; lncRNAs—long non-coding RNAs; sRNAs—small RNAs.

### 1.4. The Biology of MicroRNAs

MicroRNAs (miRNAs) are small, single-stranded, 20–25 nucleotide-long, non-coding RNAs found in humans, animals, plants, and even some viruses that are involved in post-transcriptional regulation of gene expression, primarily by silencing target messenger RNAs (mRNAs). The human genome encodes approximately 2600 pre-microRNAs (miRbase v.22, accessed 18 March 2026). Discovered initially in *C. elegans* by Victor Ambros and Gary Ruvkun (who won the 2024 Nobel Prize for this), these molecules are highly conserved across plants and animals [44,45].

miRNAs are key regulators of numerous cellular processes, including cell proliferation and development, cell differentiation, and signaling. MiRNAs bind to the 3′ untranslated region (3′UTR) of target mRNAs, thus leading to inhibition of mRNA translation or mRNA degradation. miRNA genes are transcribed using RNA polymerase II into primary miRNA (pri-miRNA). Pri-mRNAs consist of approximately one thousand nucleotides with a local stem-loop structure made of a stem, a terminal loop, and single-stranded RNA segments at both the 5′ and 3′ ends. The nuclear microprocessor complex, composed of Drosha protein and two DGCR8 proteins, processes pri-miRNA into pre-miRNA. Pre-miRNA is a molecule consisting of 65 nucleotides shaped in a hairpin and is subsequently transferred from the nucleus to the cytoplasm by exportin 5 and Ran-GTP. There, it is cleaved by a Dicer endonuclease, thus forming a mature, single-stranded miRNA. Of note, other non-canonical mechanisms and pathways of synthesis of final mature miRNA have been identified [46,47].

This mature particle is loaded into the RNA-Induced Silencing Complex (RISC). Mature miRNAs use the Argonaute (AGO) protein within the RISC to guide binding to target mRNAs, usually through partial complementarity of the 5′-miRNA end and the 3′ UTR of the target mRNA. This interaction results in mRNA silencing via destabilization (degradation) by the endonuclease AGO activity or translational repression [46,47].

**Figure 2:**
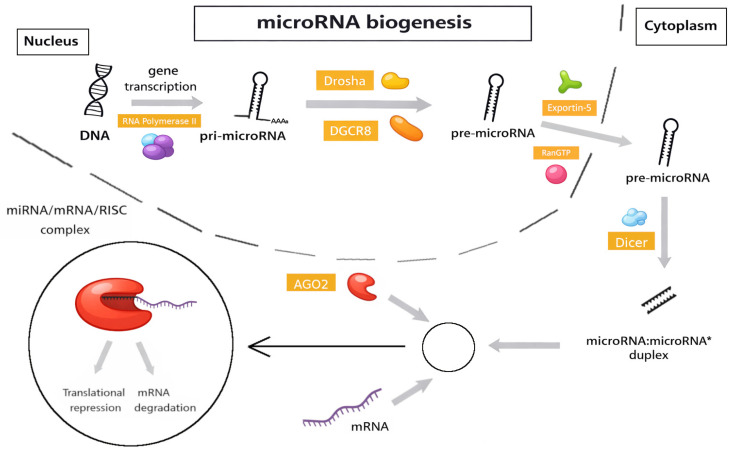
Simplified overview of canonical microRNA biogenesis.

The entire process is briefly illustrated in Figure 2.

A fascinating yet challenging issue is that a single miRNA particle can target multiple mRNAs, modulating complex gene networks. Conversely, the mRNA of a single gene can be affected by numerous miRNAs [46]. Moreover, some microRNAs affect gene expression through mechanisms such as transcriptional regulation. Single-nucleotide polymorphisms (SNPs) within the 3′UTR regions of various genes may represent a key mechanism underlying individual variability and the influence of microRNAs on various diseases, as they alter genes’ ability to bind microRNAs and thereby either inhibit or enhance this mechanism of gene expression [48].

MicroRNAs are crucial in development, cell growth, and apoptosis. Deregulation of microRNA expression is linked to many diseases, primarily different malignancies, where they function as tumor suppressors or oncogenes, as well as autoimmune diseases (e.g., rheumatoid arthritis) and different viral infections. MicroRNA particles were detected in various compartments of the human body, including bodily fluids (e.g., plasma, cerebrospinal fluid, urine), often protected within vesicles (exosomes) or associated with proteins such as AGO2, and function as intercellular messengers [47,49,50,51].

MicroRNA biogenesis begins in the nucleus, where transcription from DNA by RNA polymerase II generates a primary microRNA (pri-microRNA) with a characteristic hairpin structure. This transcript is processed by the microprocessor complex, composed of Drosha and DGCR8 (DiGeorge syndrome critical region 8), to produce precursor microRNA (pre-microRNA). Pre-microRNA is then exported to the cytoplasm in an Exportin-5/RanGTP-dependent manner. In the cytoplasm, the RNase III enzyme Dicer cleaves the pre-microRNA to generate a microRNA:microRNA* duplex, of which typically only one strand becomes functionally active. The mature microRNA is incorporated into the RNA-induced silencing complex (RISC), where it associates with Argonaute (AGO) proteins. Within the RISC, microRNA guides target recognition through partial complementarity, primarily involving the seed region (nucleotides 2–8), typically located in the 3′ untranslated region (3′ UTR) of target mRNAs. This interaction results in translational repression or mRNA degradation, thereby regulating gene expression at the post-transcriptional level [46,47].

## 2. The Role of MicroRNAs in the Pathogenesis of Primary Hypertension and Development of Hypertension-Mediated Organ Damage

Since the discovery of microRNAs, thousands of full-text manuscripts have been published on the relationship between these molecules and arterial hypertension. Studies have identified changes in microRNA expression levels in tissues, extracellular vesicles, and circulation, examined different possible pathophysiological pathways (e.g., nitric oxide synthase or renin–angiotensin–aldosterone system), and analyzed the connections between miRNAs and sex, and ethnic differences, hypertension-mediated organ damage, and virtually all possible aspects of hypertension [52,53].

Several of the most promising microRNAs that might serve as potential biomarkers for primary hypertension or be associated with its pathogenesis were presented in Table 2 [52]. It should be emphasized that, despite the numerous limitations in the use of microRNAs described later in this article, these molecules are quite stable in plasma or serum compared to other RNAs, remaining bound either to extracellular vesicles (EVs) or to proteins, primarily AGO2. MicroRNA can remain unchanged for several days at room temperature and can even survive numerous freeze–thaw cycles [53].

The origin of serum/plasma microRNA is not fully understood, though a study published in 2020 showed that plasma and serum microRNA expression patterns reflect those in liver, spleen, fatty tissue, and pericardium [54].

**Table 2:** MicroRNAs affecting blood pressure and arterial hypertension, according to [35,36], with our own modifications.

MicroRNA	Proposed Target (mRNA)	Mechanism of Action	Cardiovascular Effect
MicroRNA evaluated in the ATHENA study.			
microRNA-16	cyclins (*CCND1*, *CCND3*, *CCNE1*), *VEGFR2*, *FGFR1)*, *ADRA1A*, *VEGFA*, *BCL2*	Regulation of cell cycle and apoptosis, including endothelial and vascular smooth muscle cells and through sympathetic nervous system	Rather the anti-proliferative effect on pulmonary hypertension. The effect on systemic hypertension may be bidirectional [55,56].
microRNA-21	Mitochondrial cytochrome b, signaling pathways including *SMADs*, *FGFs/FGFRs*	Regulation of fibroblast function, cardiac cell growth, and vascular smooth muscle cell proliferation	Increase in blood pressure, development of HMOD [57,58].
microRNA-27a	*HMGCR*, *TGFBR2*	Regulation of cardiomyocyte and vascular smooth muscle cell function	Protection against hypertension and negative aortic alterations [59,60].
microRNA-27b	*FBW7*/Snail pathway, *SMAD-2/-3* pathway *HMGCR*	a “regulatory hub” in lipid metabolism,	Contradictory data on the impact on heart fibrosis, and the impact on the risk of pHTN development [61,62].
microRNA-133a	*NOTCH* signaling *SOX4*, *SOX9*	Protection against heart fibrosis and hypertrophy	Downregulated in left ventricular hypertrophy and primary hypertension [63,64].
microRNA-145	ACE, MRTF, SGRAP1, SGRAP2, SLC7A1, KLF4, KLF5, Mycocardin	Overexpression in damaged arteries (atherosclerotic plaque or aortic aneurysm), expression in vascular smooth muscle cells during embryonic development	Contradictory data may promote arterial damage or act as a compensatory factor Elevation of blood pressure [64,65].
Other important microRNAs			
microRNA-29a-3p/-29b-3p	*Lysophospholipase 1*, *VEGF-A*, *PDGFB*, *PTEN*, *TGFB2*, *SMAD3*	Nitric oxide production	Downregulation leads to an elevation in blood pressure [66].
microRNA-30a/-30b/-30c/-30d/-30e	*SMAD1*, *RUNX2*, *vimentin*	regulation of angiogenesis	microRNA-30a and -30e elevated in hypertension, -30d—lowered in hypertension [53].
microRNA-34a	*SIRT1*, *NOTCH1*, *BCL-2*, *SMAD4*, *TGF-β1*	Induction of senescent cell phenotype through downregulation of SIRT1 in endothelial and vascular smooth muscle cells	Elevated in human and animal models of hypertension and in atherosclerotic plaques [67].
microRNA-92a	*FBXW7*, *CDH1*, *NF2*, *PTEN*, *p21*, *MKK4*	Induction of endothelial and vascular smooth muscle cell injury, reduction in nitric oxide production	Elevated in people with hypertension [25].
microRNA-126	*SPRED1*, *PIK3R2*, *VCAM1*, *EGFL7*, *VEGF-A*	Influences endothelial function (e.g., inhibition of expression of VCAM1)	Conflicting results—associations found both with elevated and lowered blood pressure [68].
microRNA-192-5p	*Na-K-ATPase β1*, *ZEB-1*, *ZEB-2*	Increases sodium urinary excretion	Downregulation increases blood pressure [52].
microRNA-181a	*Renin*, *SIRT1*, *SMAD7*, *PDIA6*	Renin–angiotensin–aldosterone system	Downregulation increases blood pressure [52].
microRNA-195-5p	*NKCC2A*, *CCND1*, *CDK6*, *CDK2*	Increases sodium, potassium, and chloride tubular reabsorption in the thick ascending limb of the loop of Henle	Downregulation increases blood pressure [69].
let-7 microRNA family (let-7a, let-7b, let-7c, let-7d, let-7e, let-7f, let-7g, let-7i, mir-98, mir-202	*CCND1*, *K-RAS*, *H-RAS*, *N-RAS*, *HMGA2*	Regulation of a pro-inflammatory NF-κB pathway in endothelial cells	Elevated in people with hypertension in selected studies [25,70]

The question remains open as to how microRNAs influence blood pressure and can induce the progression from normotension to primary hypertension.

The first proposed mechanism (according to Folkov’s concept) is an effect on smooth muscle tone, thereby increasing peripheral resistance. This may be a mechanism of direct effect on VSMCs or via angiotensin II or nitric oxide. This seems to be the mechanism of action of microRNA-143, microRNA-145, microRNA-29a-3b, and microRNA-29b-3b.

The second proposed mechanism is the effect on the kidney and increased sodium retention in the renal tubule (the hypertension mechanism proposed by Guyton), and again through direct action, e.g., on the NKCC2a co-transporter (sensitive to loop diuretics) in the nephron (Henle’s) loop, and indirectly through various elements of the renin–angiotensin–aldosterone system. MicroRNA-133a, microRNA-181a, and microRNA-195-p appear to act on the kidney.

In this way, microRNAs fit into the classic concepts of the development of pHTN in humans. However, it is now believed that Guyton and Folkow’s concepts are extreme models that more closely correspond to monogenic forms of hypertension (Liddle’s syndrome on the one hand and Bilginturan’s syndrome on the other) than to the complexity of primary hypertension’s pathogenesis. pHTN can more readily be explained using Page’s modified mosaic model [12]. And in this context, microRNAs should be considered as one of the players—possibly key ones—but ones that interact dynamically with other systems (including the sympathetic nervous system and the RAA system) mentioned in Section 3.

**Figure 3:**
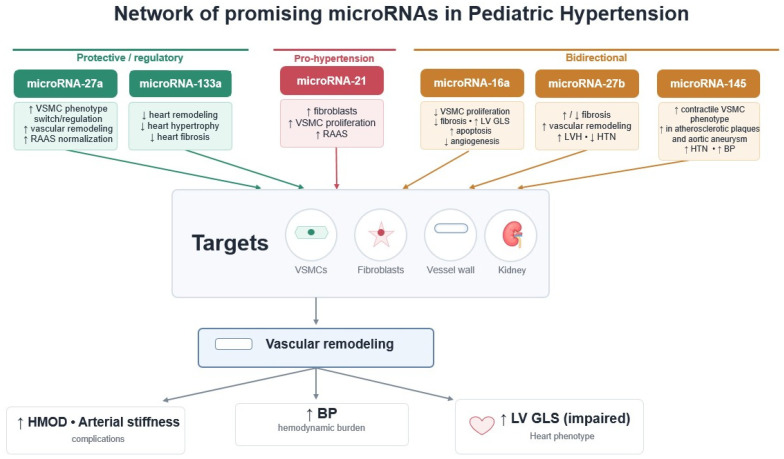
Network of selected, promising circulating microRNAs involved in pediatric hypertension, demonstrating heterogeneous and context-dependent effects on vascular smooth muscle cells, fibroblasts, vessel wall remodeling, and the renal regulatory system. These interactions contribute to vascular remodeling, hypertension-mediated organ damage (HMOD), increased blood pressure (BP), and impaired left ventricular global longitudinal strain (LV GLS). VSMCs—vascular smooth muscle cells; RAAS—renin–angiotensin–aldosterone system; LVH—left ventricular hypertrophy; HTN—hypertension.

In 2020, we launched the ATHENA (ArTerial HypErtension PAthogenesis—microRNA as a missing link in the disease development in children) project. The study aimed to evaluate the expression levels of microRNA particles in children with primary hypertension and their correlation with clinical and biochemical data, blood pressure, and hypertension-mediated organ damage (HMOD) [64]. We selected 6 molecules as promising candidates potentially involved in the regulation of blood pressure. The selection was based on data from adult studies and preceded by an in-depth analysis of the available literature. Since then, many studies have been published confirming the importance of these molecules in the development of primary hypertension and organ damage. The complex and interconnected mechanisms of action of these microRNAs are summarized in Figure 3.

### 2.1. MicroRNA-16

MicroRNA-16 is a potent suppressor of cancer growth and a key regulator in the cell cycle and is frequently downregulated in neoplasms such as chronic lymphocytic leukemia. MicroRNA-16 is located on chromosome 13q14. It inhibits cell proliferation by targeting cyclins (CCND1, CCND3, CCNE1) and CDK6, and modulates immune responses, angiogenesis, and neuronal function. MicroRNA-16 is a well-documented tumor suppressor. Reduced expression (downregulation) is observed in various malignancies, including chronic lymphocytic leukemia, prostate cancer, and ovarian cancer, often acting to stop the G1/S phase transition. Its main mechanisms of action include regulating the cell cycle by inhibiting the expression of genes that promote cell division, such as Cyclin D1 (CCND1) and CDK6. MicroRNA-16 also plays a role in inflammatory responses by improving insulin sensitivity and influencing the translation of programmed cell death ligand 1 (PD-L1), thereby affecting macrophage polarization [55,71].

This molecule seems to be involved in the development and progression of pulmonary arterial hypertension [72]. What is more, experimental data suggest that microRNA-16 may be involved in blood pressure regulation by modulating sympathetic signaling. In a mouse model, it was demonstrated that microRNA-16 directly targets the ADRA1A gene, which encodes the α1A-adrenergic receptor. Inhibiting microRNA-16 increased ADRA1A expression and was associated with reductions in systolic and diastolic blood pressure, as well as attenuating myocardial apoptosis and fibrosis [73]. This indicates a potential role for microRNA-16 in hypertensive cardiac remodeling.

Additionally, microRNA-16 has recently been linked to subclinical myocardial deformation in children. Notably, our recently published data demonstrated an association between increased circulating microRNA-16-5p and impaired left ventricular global longitudinal strain (LV GLS) in children with pHTN [56]. This suggests that microRNA-16-5p may be a potential biomarker of early subclinical myocardial dysfunction. Furthermore, previous experimental studies have demonstrated the involvement of microRNA-16 in the renin–angiotensin II system [74]. In the aforementioned study, angiotensin II was found to downregulate the expression of microRNA-16, and silencing it was found to enhance the proliferation of vascular smooth muscle cells, suggesting a protective role in vascular remodeling. This apparent discrepancy may reflect the tissue- and context-specific effects of microRNA-16, as well as its potential role as a compensatory biomarker rather than a direct mediator of pathological processes. In particular, the activation of the renin–angiotensin–aldosterone system (RAAS) in hypertension may initially lead to the downregulation of microRNA-16 at the tissue level. The observed increase in circulating microRNA-16, meanwhile, could represent a systemic compensatory response aimed at counteracting vascular remodeling and hypertrophic signaling. However, further investigation is required to confirm this.

Additionally, some reports indicate altered expression of microRNA-16 in patients with essential hypertension or hypertension-associated chronic kidney disease [53,75,76]. However, the direction and magnitude of these changes are still inconsistent across studies. Overall, the current evidence base supports the potential involvement of microRNA-16 in pathways relevant to arterial hypertension, particularly in the regulation of the sympathetic nervous system and as mentioned above, cardiovascular remodeling. However, further well-designed clinical studies are required to clarify its significance in human disease.

### 2.2. MicroRNA-21

MicroRNA-21 acts as a potent oncogene (“oncomiR”) by regulating gene expression and suppressing apoptosis. Although this molecule is frequently overexpressed in numerous cancer types, it is also becoming an increasingly significant area of interest in the study of cardiovascular and inflammatory diseases. Known as an “oncomiR,” microRNA-21 promotes tumor growth, invasion, and metastasis by targeting tumor suppressor genes, including *PTEN* and *PDCD4*. MicroRNA-21 is upregulated in most cancers, including breast, lung, and glioblastoma, and inhibits apoptosis [57].

In the cardiovascular system, microRNA-21 regulates fibroblast function, cardiac cell growth, and vascular smooth muscle cell proliferation. It was found that microRNA-21 controls numerous signaling pathways, including mTOR, NF-κB, and TGF-β1. These pathways are related to cell (also VSCM) proliferation, injury repair, and fibrosis.

Some studies have shown that microRNA-21 may play a cardioprotective role. On the other hand, other papers revealed that this particle expression level is high in patients with myocardial infarction and may act as a promoter of fibrosis and heart dysfunction. One cannot exclude the dual role of this microRNA in the pathogenesis of cardiovascular diseases, depending on the cell, body fluid, and clinical circumstances [77].

Accumulating current evidence indicates that microRNA-21 is closely involved in the pathogenesis of pHTN, particularly through its contribution to vascular dysfunction and cardiovascular remodeling [78]. MicroRNA-21 influences the TGF-β1/SMAD7 and RAAS pathways, affects the immune response, and, through these mechanisms, may be a key player in pHTN pathogenesis.

Notably, it has been demonstrated that microRNA-21 regulates key processes such as fibrosis, hypertrophy, inflammation, and cell survival [79]. Moreover, the aforementioned processes play a central role in the development of vascular stiffness and HMOD, including arterial and heart damage [63,80]. MicroRNA-21 negatively affects VSMC biology. Proatherogenic stimuli induce microRNA-21 expression in VSMCs and their differentiation into lipid-accumulating cells [81]. Not to mention, microRNA-21 overexpression was found in atherosclerotic plaques [67] and correlated with common carotid artery intima-media thickness (cIMT) [58].

A recent study showed higher levels of microRNA-21 expression in individuals with resistant hypertension compared to normotensives and newly diagnosed hypertensives. In this study, the expression of microRNA-21 correlated positively with blood pressure and aldosterone concentration [82]. Another study has shown that circulating levels of microRNA-21 are correlated with aldosterone, blood pressure, and HMOD, such as albuminuria [83]. These findings further support that microRNA-21 expression reflects the activation of the RAAS, hemodynamic load, and early HMOD in pHTN. Interestingly, a meta-analysis including 12 articles, involving 546 cases and 436 controls, on the relationship between microRNA-21 and arterial hypertension was published. The pooled results revealed that the expression levels of microRNA-21 were significantly elevated in hypertensives compared with normotensives [84]. Unfortunately, there are still some conflicting data. A prospective 5-year Japanese study failed to demonstrate that microRNA-21 predicts the development of pHTN [59].

Considering the above, elevated circulating levels of microRNA-21 have been associated with disease severity and adverse cardiovascular outcomes, supporting its potential as a biomarker reflecting ongoing vascular injury and remodeling. Therefore, microRNA-21 may serve not only as a mechanistic regulator but also as a clinically relevant marker for monitoring disease progression and early cardiovascular involvement, perhaps in pediatric patients with pHTN as well.

In summary, microRNA-21 appears to be closely associated with pHTN and may be a promising biomarker. This may be the most important microRNA molecule in this disease entity. Prospective and interventional studies are undoubtedly needed.

### 2.3. MicroRNA-27a and MicroRNA-27b

MicroRNA-27a and microRNA-27b belong to a larger microRNA cluster (microRNA-23a~27a~24-2) located on human chromosome 19 and chromosome 9 (C9orf3 gene, 9q22.1), respectively. This cluster is a crucial post-transcriptional regulator of gene expression, involved, e.g., in cancer biology [85]. MicroRNA-27a seems to act alongside microRNA-21, as a potent oncogene (“oncomiR”) or, inversely, may suppress tumor growth, depending on the specific tissue and cancer type. It promotes proliferation and invasion in cancers such as gastric, breast, ovarian, and laryngeal carcinoma. On the other hand, it can inhibit growth and metastasis in renal cell carcinoma, esophageal squamous cell carcinoma, and colon cancer. In addition, microRNA-27a is a critical regulator of cholesterol biosynthesis by targeting the *HMGCR* gene. It also negatively regulates adipocyte (fat cell) differentiation by suppressing PPARγ. In addition, microRNA-27a is essential for maintaining bone homeostasis. It functions as a negative regulator of osteoclast differentiation; its loss can lead to severe osteoporosis [86,87].

Like microRNA-27a, microRNA-27b plays an essential role in regulating lipid homeostasis and is often referred to as a “regulatory hub” in lipid metabolism. The expression level of microRNA-27 b is commonly altered in various forms of dyslipidemia [61].

MicroRNA-27a and microRNA-27b have also numerous potential roles in the cardiovascular system. MicroRNA-27a regulates cardiac function [88] and cardiomyocyte apoptosis [89], and also affects vascular smooth muscle cell proliferation and migration [90]. An experimental Chinese study showed that exercise training in spontaneously hypertensive rats increased microRNA-27a levels in the aortas, restoring disturbed renin–angiotensin–aldosterone system biology and, at least in part, explaining the protective effect of exercise on aortic function and structure [60]. Suzuki et al. reported that the expression level of circulating microRNA-27a may predict the occurrence of hypertension (“a negative biomarker”). Serum microRNA-27a was significantly lower in newly hypertensive subjects than in normotensive subjects [59].

As for microRNA-27b, it can promote myocardial fibrosis by modulating the FBW7/Snail pathway [62]. Conversely, another study revealed that microRNA-27b ameliorated atrial fibrosis and atrial fibrillation by inactivating the Smad-2/3 pathway via targeting *ALK5*. The latter results suggest an anti-fibrotic potential of this molecule in the heart. It was even proposed as a target for the therapies of heart failure [91]. An interesting human study showed that serum microRNA-27b expression levels were elevated in subjects with AH and left ventricular hypertrophy (LVH) compared to hypertensive patients without LVH and in normotensives. The authors concluded that the expression level of microRNA-27b is a potential biomarker for left ventricular hypertrophy [92].

DROSHA is a class 2 ribonuclease III enzyme, which, together with DGCR8, is involved in an initial step in microRNA processing. DROSHA participates in many other pathways of RNA maturation and degradation. DROSHA plays a role in vascular smooth muscle cell proliferation and differentiation, thus potentially influencing arterial tonus and blood pressure [93]. The SNP rs10719, located within microRNA-27b, is associated with the risk of pHTN. Chinese authors reported that rs10719 prevents the interplay between microRNA-27b and DROSHA, which may underlie the association between this SNP and the risk of pHTN [94].

### 2.4. MicroRNA 133a

MicroRNA-133a is encoded by two genes, miR-133a-1 and miR-133a-2, which are placed on chromosomes 18 and 20. It is a muscle-enriched (“myomiR”) that regulates cardiac and skeletal muscle development and also inhibits the growth of various cancers. The expression of microRNA-133a is very high in the myocardium and is crucial for heart development and function, where it suppresses cardiomyocyte apoptosis and regulates gene expression [95]. It is downregulated during heart hypertrophy and acts as an anti-apoptotic factor in damaged cardiomyocytes, making it a potential therapeutic target. Also, a MicroRNA-133a/b complex, by influencing the Notch signaling pathway, promotes atherosclerotic plaque formation and adverse alterations (e.g., fibrosis) in the myocardium [63].

The study by Akerman revealed elevated plasma expression levels of microR-133a in individuals with hypertension compared to those with normal blood pressure [96]. Also, Koval et al. investigated plasma microRNA-133a levels in patients with primary hypertension. In their study, plasma microRNA-133a levels were lower in patients with AH than in healthy individuals. The authors have also reported significantly diminished microRNA-133a expression levels in the plasma of subjects with LVH compared with patients with normal heart structure. The authors identified no additional determinants (e.g., comorbidities) of plasma microRNA-133a expression levels. The authors concluded that reducing plasma levels of miR-133a is essential in the development of pHTN and in the pathological remodeling of the heart [97]. In another study by the same group analyzing hypertensive patients, the expression of microRNA-133a was decreased in patients with left ventricular diastolic dysfunction. It was negatively correlated with left ventricular mass index [98].

Kontaraki et al. unmasked similar results. They also reported that patients with arterial hypertension had diminished microRNA-133a expression (5.06 ± 0.50 vs. 13.20 ± 2.15, *p* < 0.001) than healthy controls. In hypertensive patients, a significant negative correlation was observed between miR-133a expression levels and the indexed left ventricular mass (r = −0.431, *p* < 0.001) [99].

Furthermore, in our previous study, changes in microRNA-133a expression after exercise were positively correlated with LVMI [64]. However, this association was not observed in the full cohort. Our currently unpublished preliminary data also suggest that children with pHTN have lower baseline and post-exercise levels of microRNA-133a, along with a blunted exercise-induced response, compared to healthy controls. The results also showed a positive correlation between microRNA-133a expression and BMI Z-scores, as well as measures of adiposity (waist and hip circumference). This indicates a potential, albeit currently limited, role for miR-133a as a biomarker of myocardial hypertrophy in pHTN.

### 2.5. MicroRNA-145

The primary role of microRNA-145 is to suppress tumor growth by regulating the expression of genes such as *c-MYC*. It acts as a molecular brake, often reducing the expression of proteins that promote cancer progression, such as MUC1, or stemness, such as OCT4 [100,101].

Beyond cancer, microRNA-145 is the most abundant microRNA in vascular smooth muscle cells (VSMCs), essential for maintaining their stability and playing an important role in VSMC differentiation. The expression of microRNA-145 is also found in plasma and the endothelium, highlighting its significance in the cardiovascular system. MicroRNA-145 targets crucial pathways and molecules in the cardiovascular system, including angiotensin-converting enzyme (ACE) or myocardin-related transcription factor B [102]. Additionally, microRNA-145 plays a central role in maintaining the contractile phenotype of VSMCs by regulating key transcription factors, including KLF4, myocardin, and SRF [103]. A loss of microRNA-145 expression can lead to a switch in phenotype from contractile to synthetic and proliferative, which contributes to vascular remodeling, increased arterial stiffness, and impaired regulation of vascular tone [104,105]. Thus, these mechanisms directly link microRNA-145 dysregulation to blood pressure control and the development of hypertension.

From more clinical perspective, Santovito et al. examined material obtained during endarterectomy from patients with AH and found very high levels of microRNA-145 expression in atherosclerotic plaques [106]. Experimental data suggest that microRNA-145 might play a harmful role in the pathogenesis of both systemic and pulmonary hypertension [107].

Another experimental study investigated the microRNA-145 in spontaneously hypertensive rats (SHR). The expression of microRNA-15 in the aorta was higher in the SHR group than in healthy animals. In rat vascular endothelial cells (RVECs), in which microRNA-145 was silenced, the expression of solute carrier family 7 member 1 (SLC7A1) and phosphorylated endothelial nitric oxide synthase rose. A dual-luciferase reporter assay confirmed the hypothesis that SLC7A1 is directly targeted by microRNA-145. The authors concluded that microRNA-145 might be a crucial player in the development of arterial hypertension.

In our group, microRNA-145 showed a distinct pattern, suggesting a possible beneficial effect in children with pHTN [64]. In our study, the expression of this microRNA increased after a single exercise session and was negatively correlated with blood pressure and HMOD. The explanation of our findings may be found in an experimental study by Li et al. The authors revealed that microRNA-145 negatively regulated the production of GATA-binding protein 6 (GATA6), thereby attenuating cardiomyocyte hypertrophy in the experimental model [65].

Our results are consistent with Özkan’s study. The authors found that downregulation of microRNA-145 was an independent predictor of arterial hypertension. Renalase concentration also showed a positive correlation with microRNA-145 expression [108].

## 3. Potential Therapeutic and Diagnostic Implementations

Non-coding RNAs can be a new therapeutic measure in the treatment of both rare and common diseases. The use of antisense RNA—interfering with mRNA (small interfering RNA)—allows protein production to be inhibited despite increased gene expression. SiRNA-based drugs are now approved medications for many rare entities, including primary hyperoxaluria type 1 [109]. Clinical trials are also conducted in hypertensive patients. Zilebesiran is a siRNA that targets angiotensinogen mRNA, thereby inhibiting its hepatic synthesis for up to half a year after a single injection. Once animal studies confirmed its safety and efficacy comparable to those of oral RAS blockers, human clinical trials were conducted.

The study of zilebesiran in humans had 3 parts. In the first part, hypertensive subjects randomly received either a single subcutaneous injection (doses from 10 to 800 mg) or a placebo and were observed for 24 weeks. Another part evaluated the efficacy of the 800 mg single dose of zilebesiran on blood pressure, depending on salt intake. In the final part, participants received the 800 mg zilebesiran injection in combination with irbesartan. The drug was effective in lowering blood pressure for a whole 24-week period with only mild side effects (transient injection-site reactions). No patients experienced hypotension, hyperkalemia, or deterioration in kidney function [110]. Elevated RAAS activity is crucial in certain clinical circumstances, such as hypovolemic shock. For these situations, a REVERSIR technology was developed. REVERSIR uses short, high-affinity, single-stranded oligonucleotides designed to reverse siRNA-mediated RNA interference in vivo [79] rapidly. We are deeply convinced that siRNA technology could revolutionize antihypertensive therapies, especially for non-adherent patients, thereby substantially reducing the impact of non-adherence on health care costs [111].

## 4. Gaps in Current Knowledge

While microRNA research has advanced significantly—highlighted by the 2024 Nobel Prize in Physiology or Medicine—several critical knowledge gaps and technical bottlenecks remain. Firstly, many discovered microRNAs lack specific biological functions and the gene networks they regulate. Also, there is a general lack of data on microRNA expression at the cell level. Researchers often do not know if a microRNA and its predicted target are ever present in the same cell at the same time. In addition, many circulating microRNAs identified as “biomarkers” are not disease-specific (e.g., hypertension-specific). The same microRNA may be up- or downregulated in multiple unrelated conditions and in response to different stimuli, e.g., food or exercise. The results of the microRNA studies on the same entity are highly inconsistent, often due to small sample sizes, varying subject characteristics, and different isolation and evaluation protocols. As miRNAs are found in the nucleus, mitochondria, and endoplasmic reticulum, the precise mechanisms by which they are transported to these organelles remain unclear. It also remains an open question whether the specific functions of individual microRNAs are conserved across species, particularly in non-model organisms. Standardizing detection for short, highly homologous sequences remains difficult. Current methods are prone to bias during isolation and struggle with single-nucleotide specificity [50,112].

Finally, there are numerous bioinformatic limitations. Namely, analyzing the relationships between hundreds of candidate microRNAs and their targets is often impossible. There is a lack of integrated, automated software that efficiently extracts these functions from complex gene networks.

## 5. Limitations of Using MicroRNA in Clinical Practice as Biomarkers or Therapeutic Targets

Using microRNAs as disease markers or therapeutic goals holds significant potential, but numerous limitations hamper their translation into everyday clinical practice. These include technical challenges in measurement, lack of standardization, poor target specificity, and issues with delivery and stability [113].

First, to reliably select biomarkers for diseases such as pHTN, one should not preselect based on a single study; rather, conduct a preliminary profiling analysis. Such an analysis should be carried out on a smaller group (a pilot study) comprising at least 10 individuals, including both those with and without hypertension. Based on the results obtained, potential microRNAs should be selected for verification by real-time quantitative reverse transcription polymerase chain reaction (qRT-PCR) in two large groups (hypertensive vs. normotensive), with sample sizes estimated using statistical methods [53].

While miRNAs are rather stable in biofluids (serum, plasma, urine), several obstacles prevent their routine use as biomarkers. First, pre-analytical variability represents a major source of bias. Circulating miRNA levels are highly sensitive to sample collection, processing, and storage conditions. There is no consensus on protocols for sample type (serum, plasma, etc.), sample collection, handling, storage, and isolation. These issues lead to significant variability and discrepancies across published studies. Further limitations include the lack of standardization in RNA isolation and quantification methods. A variety of extraction kits, protocols and input volumes can produce inconsistent results. Different quantification methods (qRT-PCR, RNA sequencing, microarrays) often yield opposite results. In addition, there is a lack of universally accepted normalizers (internal controls) for measuring circulating microRNA levels. Although endogenous reference miRNAs are frequently utilized in studies of tissue and cells, their stability in plasma or serum remains uncertain. Consequently, many studies rely on exogenous spike-in controls to monitor extraction and reverse transcription efficiency. However, these controls do not account for biological variability between samples. Alternative approaches have been proposed, such as global mean normalization or normalization to plasma volume, but none have been universally accepted. It should be noted that global mean normalization usually necessitates the measurement of a sufficiently large number of miRNAs to ensure stable estimation of the average expression level, which could restrict its applicability in studies focusing on a small panel of selected miRNAs.

Notably, the analytical validation of RT-qPCR assays is frequently inadequately documented. Key parameters such as amplification efficiency, dynamic range, and intra- and inter-assay variability are not consistently evaluated, nor is the presence of PCR inhibitors. This may limit the interpretability of quantitative results. The validity of relative quantification methods, such as the 2^ΔΔCq^ approach, depends on the assumption that amplification efficiencies are comparable between targets and reference controls, a factor that is not always verified.

Furthermore, the biological variability and heterogeneity of circulating microRNAs complicate final analysis and conclusions. These molecules originate from multiple tissues and are present in various forms, such as protein-bound fractions and extracellular vesicles, potentially reflecting distinct biological processes. Consequently, differences observed between studies may be influenced by disease-related mechanisms and methodological inconsistencies.

Finally, many reported microRNA biomarkers are not disease-specific and are frequently detected in multiple, different, or overlapping entities. For instance, as already mentioned, microRNA-21 is upregulated in numerous cancers (colorectal, lung, breast, prostate, etc.), and in cardiovascular diseases, which limits its value as a disease-specific biomarker. Additional factors, such as hemolysis, may falsely elevate plasma or serum microRNA levels, as many microRNAs are abundant within cells. Patient-related factors such as age, sex, ethnicity, diet, comorbidities, and medications can significantly affect circulating microRNA expression levels [114,115].

Given that in adults, hypertension is rarely the only condition affecting a patient and often occurs in conjunction with metabolic syndrome, atherosclerosis, and, not infrequently, type 2 diabetes and chronic kidney disease, it is difficult to unequivocally demonstrate a link between a specific microRNA and elevated blood pressure alone. Therefore, it seems crucial to study a population as free of comorbidities as possible. In this regard, the ATHENA study, which reliably assesses the expression of selected microRNAs in children and adolescents with primary hypertension—with secondary forms, CKD, and DM excluded—and includes a detailed evaluation of clinical data and HMOD, offers hope for identifying genuine associations between microRNAs and primary hypertension [64].

Although siRNAs (RNA-based therapeutics that induce sequence-specific silencing of target mRNA) are slowly entering clinical practice, it is not as easy with drugs that affect microRNA. Developing microRNA-targeted medications faces numerous challenges. Because a single miRNA particle targets numerous mRNAs, therapeutic modulation of a single miRNA-based drug may cause unintended, broad-spectrum gene silencing in non-target tissues, leading to unforeseen sequelae. Delivering microRNAs to specific tissues (e.g., kidneys, heart, or cancer cells) remains a major bottleneck. Unmodified microRNAs are rapidly degraded by nucleases in the bloodstream and are cleared by the kidneys. In addition, some microRNA-based treatments have been found to trigger an immune response, leading to the activation of inflammatory cytokines. Finally, many miRNA inhibitors or mimics show really moderate efficacy in early clinical trials, with none, for example, having progressed to Phase III by now [116,117].

## 6. Conclusions

Recent years have brought significant progress in research into the pathogenesis of primary hypertension on the one hand, and the role of small non-coding RNAs, including microRNAs, in numerous human pathologies on the other. While the contribution of individual genes does not appear significant, post-transcriptional changes in microRNAs may open a new avenue of research into primary hypertension. Although the current evidence on microRNAs in pediatric primary hypertension is inconsistent, this variability likely reflects their context- and tissue-specific roles rather than a lack of biological relevance. In this context, microRNAs can be considered not only as promising biomarkers, but also as dynamic regulators of the vascular, renal, and cardiac pathways involved in blood pressure control. Our review highlights their dual role as both potential biomarkers and context-dependent regulators, which may explain the inconsistencies observed across studies.

Thus, microRNAs can be both valuable biomarkers and therapeutic targets. The most promising molecules have already been selected. However, the pleiotropic and context-dependent effects of microRNAs—where they may exert beneficial actions in one tissue while being detrimental in another or have different roles across disease stages—make identifying the appropriate therapeutic target in the right place and at the right time a key challenge for future research.

In our opinion, studying children is particularly valuable, as there are fewer environmental factors to consider that have accumulated during their lifetime, making it easier to derive conclusions. This makes it easier to identify microRNA involvement in the development of diseases such as primary hypertension. Future research should focus on standardized methodologies, larger, well-characterized pediatric cohorts, and longitudinal studies, to better define the clinical utility and translational potential of microRNAs in primary hypertension.

## Figures and Tables

**Table 1:** Selected mechanisms involved in the pathogenesis of primary hypertension in children and adolescents and the potential role of microRNAs.

Mechanism	Description/Pathophysiology	Possible MicroRNA Involved
Increased sodium sensitivity	Excessive renal tubular sodium reabsorption [13] (not specific for children)	MicroRNA-19 [14] and microRNA-RNA-485 [15]
Sympathetic nervous system overactivity	Sympathetic overdrive often associated with obesity and metabolic syndrome [16] (not specific for children but may be particularly important in the pediatric population)	MicroRNA-146a/b [17], microRNA-155 [17] and microRNA-181a [18]
RAAS overactivation	Increased activity of the renin–angiotensin–aldosterone system both systemically and locally, leading to blood pressure elevation and, e.g., left ventricular hypertrophy [19,20] (not specific for children)	Numerous micro-RNAs (e.g., microRNA-16 [21], microRNA-21 [21,22], microRNA-133a [21,23])
Altered body composition	Increased fat mass with relative deficiency of lean body mass and skeletal muscle (sarcopenia) [24] (not specific for children but may be particularly important in the pediatric population)	Numerous micro-RNAs (e.g., microRNA-21, microRNA-27a) [25]
Early vascular aging	Accelerated biological aging with early vascular changes, including increased arterial stiffness and wall thickening (not specific for children but may be particularly important in the pediatric population) [26]	Numerous micro-RNAs (e.g., microRNA-103, microRNA-107, microRNA-128) [27]
Immune system activation	Chronic low-grade inflammation and immune system overactivation leading to blood pressure elevation and formation of hypertension-mediated organ damage [28,29] (not specific for children)	MicroRNA-126 [30], microRNA-130a [30], microRNA-146 [30] and microRNA-199a [31]

## Data Availability

No new data were created or analyzed in this study.
